# A randomized sequential allocation study on the optimum programmed intermittent epidural boluses interval time with different concentrations of ropivacaine combined with the dural puncture epidural technique for labor analgesia

**DOI:** 10.3389/fphar.2024.1508514

**Published:** 2025-01-07

**Authors:** Jingjing Mao, Yi Chen, Linsen Sun, Xiaoxiao Xu, Kai Xu, Tingting Ren, Xiangsheng Xiong, Weibing Zhao

**Affiliations:** ^1^ Department of Anesthesiology, Huai’an Hospital Affiliated to Yangzhou University (The Fifth People’s Hospital of Huai’an), Huaian, China; ^2^ Department of Anesthesiology and Perioperative Medicine, General Hospital of Ningxia Medical University, Yinchuan, China; ^3^ Yangzhou Institute of the Heart and Great Vessels (YIHGV), Yangzhou University, Yangzhou, China

**Keywords:** interval time, programmed intermittent epidural boluses, different concentrations, ropivacaine, dural puncture epidural

## Abstract

**Background:**

The combined technique of programmed intermittent epidural boluses (PIEB) and dural puncture epidural (DPE) is currently considered a more effective mode for labor analgesia. We investigated the optimal interval time for PIEB administration with different concentrations of ropivacaine combined with the DPE for labor analgesia.

**Methods:**

Ninety patients with cervical dilation of <5 cm and a VAS score >5 were randomly assigned to receive labor analgesia with ropivacaine at concentrations of 0.075% (0.075% group), 0.1% (0.1% group), and 0.125% (0.125% group). In each group, an initial administration of a combination of ropivacaine 12 mL and sufentanil 0.3 μg/mL was followed by an additional dose of ropivacaine 10 mL and sufentanil 0.3 μg/mL after 30 min. The initial PIEB interval time was set at 40 min for the first patient in each group, and subsequent interval times for the following patients were adjusted based on meeting analgesic needs (VAS score ≤1) with a gradient of 10 min. The primary outcome was the ED90 of interval time required to achieve analgesic needs during PIEB with different concentrations of ropivacaine, employing an up-and-down sequential allocation method.

**Results:**

The optimal PIEB interval times for ropivacaine concentrations of 0.075%, 0.1%, and 0.125% were determined to be 40.9 (95% CI, 35.3–45.8), 45.3 (95% CI, 39.3–51.5), and 52.9 (95% CI, 46.8–59.3) minutes respectively, while comparable maternal and neonatal outcomes were observed across all groups.

**Conclusion:**

When PIEB is combined with DPE for labor analgesia, the optimal PIEB interval times for ropivacaine concentrations of 0.075%, 0.1%, and 0.125% were determined to be 41, 45, and 53 min respectively.

## 1 Introduction

Labor pain has always been a critical factor that affects patients’ childbirth experience. Epidural analgesia, being the most frequently used and effective form of labor pain relief, plays an essential role in reducing labor pain (Meng et al., 2023). In comparison to non-epidural analgesia, it significantly alleviates pain in patients without elevating the incidence of adverse events and enhances their satisfaction (Wang et al., 2017). The utilization rate of epidural analgesia in developed countries can reach approximately 90% (Brebion et al., 2021). With the implementation of large-scale activities such as No Pain Labor and Delivery, there has also been substantial improvement in China’s adoption rate of epidural analgesia (Drzymalski et al., 2021).

With the advancement of research on epidural labor analgesia, the primary focus lies in optimizing analgesic methods, including various types of local anesthetics, drug concentration and dosage, as well as techniques such as compined spinal-epidural (CSE), programmed intermittent epidural boluses (PIEB), continuous epidural infusion or dural puncture epidural (DPE) (Guasch et al., 2020). The ultimate objective is to effectively alleviate pain, enhance satisfaction, and minimize adverse events such as motor blockade, conversion to cesarean section, and instrumental delivery (Halliday et al., 2022a). Motor blockade can be avoided by reducing the concentration of local anesthetics and utilizing medications with potent analgesic effects while exerting minimal impact on motor function; for instance, ropivacaine (Lee et al., 2004). Currently, both ropivacaine and bupivacaine are efficacious for epidural labor analgesia, with low concentration combined with opioids being recommended to optimize labor analgesia (Practice Guidelines, 2016). Studies have reported the effectiveness of ropivacaine combined with opioids at concentrations of 0.075% (Owen et al., 2002), 0.1% (Boselli et al., 2004; Fischer et al., 2000) and 0.125% (Fidkowski et al., 2019; Mukherjee et al., 2013) for epidural labor analgesia (Shi et al., 2024; Lee et al., 2004).

Additionally, the combined technique of PIEB and DPE is currently considered a more effective mode for labor analgesia (Yao et al., 2023). The advantage of PIEB lies in its ability to provide equivalent analgesia levels as continuous epidural infusion, while minimizing motor blockade and reducing local anesthetics usage without increasing maternal and neonatal adverse events (Ojo et al., 2020; Song et al., 2021). DPE currently stands as the most effective analgesic technique due to its mechanism, in which the local anesthetic in the epidural space enters the subarachnoid space through puncture. This results in a faster onset of analgesia and improved reduction of asymmetric block, ensuring greater stability (Wang et al., 2021).

The investigation of the optimal interval time for PIEB administration with different concentrations of ropivacaine combined with DPE for labor analgesia is of significant importance in optimizing analgesic efficacy, enhancing labor experience, and ensuring maternal safety by considering potential impacts on motor blockade. Thus, we investigated the optimal interval time for PIEB administration with different concentrations of ropivacaine combined with the DPE for labor analgesia.

## 2 Methods

This randomized sequence allocation study was approved by the Ethics Committee of the Huai’an Hospital Affiliated to Yangzhou University (The Fifth People’s Hospital of Huai’an) prior to the implementation of the study (No. HAWY-KY-2023–016-01) and registered on Chinese Clinical Trials (No. ChiCTR2300072345). The study design adhered to the guidelines of the Consolidated Standards of Reporting Trials (CONSORT) and the Helsinki Declaration, and it was conducted from July 2023 to July 2024. Informed consent was obtained from all participating patients prior to enrollment. Patients aged 18–40 with a singleton pregnancy, cervical dilation of <5 cm, VAS score >5, and an American Society of Anesthesiologists physical status I-II were enrolled. Patients with hypertension (including chronic hypertension, gestational hypertension, and preeclampsia), gestational diabetes, a BMI ≥40 kg/m^2^, fetal distress, fetal macrosomia, other analgesic methods, or contraindications such as epidural analgesia, allergy to ropivacaine or sufentanil were excluded.

The patients underwent regular uterine contractions and were assessed by a gynecologist, a midwife, and an anesthesiologist prior to entering the labor analgesia protocol. All patients receive standardized mother-baby care. Upon admission to the delivery room, maternal vital signs were continuously monitored, including electrocardiogram (ECG), non-invasive blood pressure (NBP), and oxygen saturation (SpO_2_), while continuous fetal heart monitoring was also conducted. An 18G needle was inserted into the maternal upper arm for intravenous fluid administration. Before administration of labor analgesia, the patients were divided into three groups with different concentrations of ropivacaine: 0.075% (0.075% group), 0.1% (0.1% group), and 0.125% (0.125% group). The random sequences were generated using SPSS software in a 1:1:1 ratio, and the assignments were placed in opaque sealed boxes. Both the patients and the anesthesiologist responsible for administering labor analgesia were blinded to the grouping allocation. Another anesthesiologist (evaluator), who was aware of the grouping but not involved in labor analgesia management, prepared the drugs required for each group by diluting them with normal saline solution. Only the drug name was labeled on the syringe and electronic infusion pump, while no information regarding drug concentration was provided.

The administration of labor analgesia was performed by three experienced anesthesiologists. After insertion into the L2-3 epidural space, a 25G needle punctured the arachnoid membrane (without administering local anesthetic), and an epidural catheter was placed with a depth of 4 cm in the epidural space. After ensuring the absence of excessive subarachnoid space or intravenous injection of local anesthetic, an initial dose of ropivacaine 12 mL + sufentanil 0.3 μg/mL was administered through the epidural catheter based on the assigned groups (0.075% group, 0.1% group or 0.125% group) following a 5-min observation period. In the PIEB protocol, ropivacaine 10 mL + sufentanil 0.3 μg/mL was added after 30 min from the initial loading dose, and the PIEB interval time was determined using a sequential allocation method for subsequent patients. The initial PIEB interval time for the first patient was set at 40 min, while for subsequent patients, it was adjusted based on whether the current interval time could meet their analgesic needs (VAS score ≤1). The gradient for adjusting the PIEB interval time was set at 10 min. If the current interval time adequately addressed the patient’s analgesic needs until full cervical dilation occurred, then the subsequent patient would have an increased gradient of 10 min; conversely, if these needs were not met, then the gradient would decrease by 10 min. The rescue bolus was administered when the VAS score reached ≥3, 30 min after the initial labor analgesia. Depending on their respective group assignments, a dose of ropivacaine 10 mL + sufentanil 0.3 μg/mL was given. If the patient’s VAS score remains ≥3 after receiving the rescue bolus, it is considered a failure in labor analgesia and labor analgesia will be restarted while withdrawing from the study. The Patient-Controlled Epidural Analgesia (PCEA) protocol consisted of each dose containing 5 mL of ropivacaine +0.3 μg/mL sufentanil, with a lock-in time of 20 min.

The primary outcome was the ED90 of interval time required to achieve analgesic needs, as indicated by a VAS score ≤1, during PIEB with different concentrations of ropivacaine using an up-and-down sequential allocation method. Secondary outcomes included pain-related measures, such as VAS scores (ranging from 0 to 10 points; with 0 indicating no pain and 10 indicating unbearable pain), time taken for analgesia to achieve a VAS score ≤1 after inital dose of 12 mL ropivacaine +0.3 mcg/mL sufentanil, proportion of VAS scores ≤1 within 10 min, sensory block (left, right, and highest), proportion of asymmetric sensory block (>2 dermatome levels difference in sensory block between the left and right sides), proportion of block level > T6, proportion of S1 and S2 blocks, number of rescue bolus administrations, and PCEA. Time intervals for assessing VAS scores were at baseline and at 10 min, 20 min, 30 min, 1 h, 2 h, 3 h, 4 h, 5 h, and 6 h after lobar analgesia. The additional assessment included the Bromage score, which was evaluated within 30 min following the initiation of labor analgesia and graded as follows: 0 points for knee and ankle fully bent; 1 point for knee partially bent and ankle fully bent; 2 points for inability to bend the knee but ankle partially bent; and 3 points for inability to bend both knees and ankles. Other parameters assessed included the proportion of motor blockade, *postpartum* headache, and conversion to cesarean section, total analgesia time, Apgar scores at 1 and 5 min, as well as maternal satisfaction (overall assessment was conducted *postpartum* and rated on a scale of 1-5 with higher scores indicating greater satisfaction).

### 2.1 Statistical analysis

The sample size was determined based on previously reported literature, where 20–40 cases were considered sufficient for sample size analysis using the up-and-down sequence allocation method. Therefore, we included 30 patients in each group for final analysis (Wang et al., 2021).

The normal distribution of continuous variables was assessed using the Kolmogorov-Smirnov test. For comparing groups that exhibited a normal distribution, a one-way analysis of variance (ANOVA) was utilized. In case of significant differences among the groups, a Bonferroni test was conducted for pairwise comparisons. For groups that deviated from a normal distribution, a Kruskall-Wallis test was utilized to compare among the groups, and if there were significant differences observed, a Dunn test was conducted for pairwise comparisons. Categorical variables were compared using the chi-square test. If there was a statistically significant difference observed among the groups in the chi-square test, paired tests would be conducted between each pair of groups. Repeated measurement indicators such as VAS scores were analyzed using repeated measures ANOVA. The ED90 of the optimal PIEB interval time for three concentrations was examined through isotonic regression analysis. Data analysis was performed using IBM SPSS Statistics version 25.0 (IBM SPSS, Inc., Chicago, IL). A significance level of *P* < 0.05 indicated statistically significant differences.

## 3 Results

**FIGURE 1:**
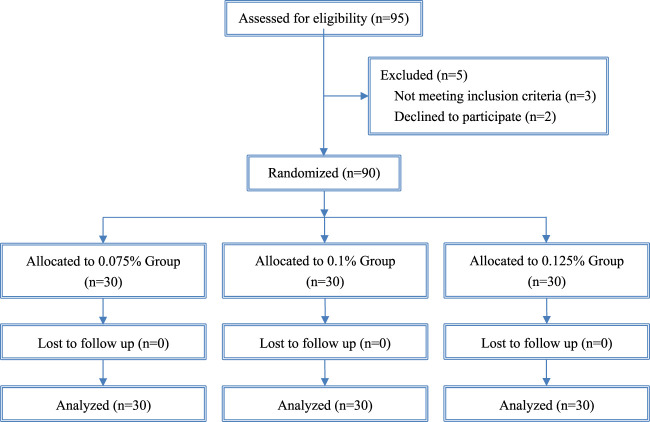
Study flow diagram.

A total of 90 patients (30 in each group) were included in the final analysis, and Figure 1 shows the inclusion flowchart. There were no significant differences in maternal characteristics among groups, as presents in Table 1.

**TABLE 1:** Maternal characteristics.

	0.075% group (n = 30)	0.1% group (n = 30)	0.125% group (n = 30)	*P* value
Age, years	27.80 ± 5.59	26.83 ± 4.41	27.30 ± 5.94	0.784
Height, kg	160.70 ± 5.43	162.77 ± 4.51	161.90 ± 4.63	0.262
Weight, cm	70.47 ± 10.06	74.75 ± 13.46	73.43 ± 10.03	0.328
Body mass index, kg/m^2^	27.32 ± 3.87	28.17 ± 4.75	27.97 ± 3.39	0.693
Gestational age, (weeks)	40 [39, 40]	39 [38, 40]	39 [38, 40]	0.212
Unipara, n (%)	17 (56.67)	21 (70.00)	16 (53.33)	0.378
Spontaneous labor, n (%)	18 (60.00)	13 (43.33)	22 (73.33)	0.061
Cervical dilation, cm	2 [2, 2]	2 [2, 2]	2 [2, 3]	0.210
Baseline maternal SBP, mmHg	120.60 ± 10.29	119.70 ± 10.94	123.53 ± 8.06	0.293
Baseline maternal DBP, mmHg	73.73 ± 6.41	73.47 ± 7.06	75.67 ± 6.27	0.373
Baseline maternal MAP, mmHg	89.35 ± 7.23	88.88 ± 7.28	91.62 ± 5.82	0.254
Baseline maternal HR, bpm	81.60 ± 7.07	82.60 ± 9.85	83.10 ± 8.13	0.782
Baseline fetal HR, bpm	139.70 ± 11.44	140.10 ± 7.64	141.90 ± 6.45	0.587
First stage of labor, min	350 [300, 400]	350 [300, 550]	400 [300, 450]	0.804
Second stage of labor, min	30 [20, 50]	20 [15, 40]	25 [20, 35]	0.381
Prolonged second stage, n (%)	0 (0.00)	0 (0.00)	0 (0.00)	>0.999

Values are mean ± SD, or median (IQR). SBP, systolic blood pressure; DBP, diastolic blood pressure; MAP, mean arterial pressure; HR, heart rate.

**FIGURE 2:**
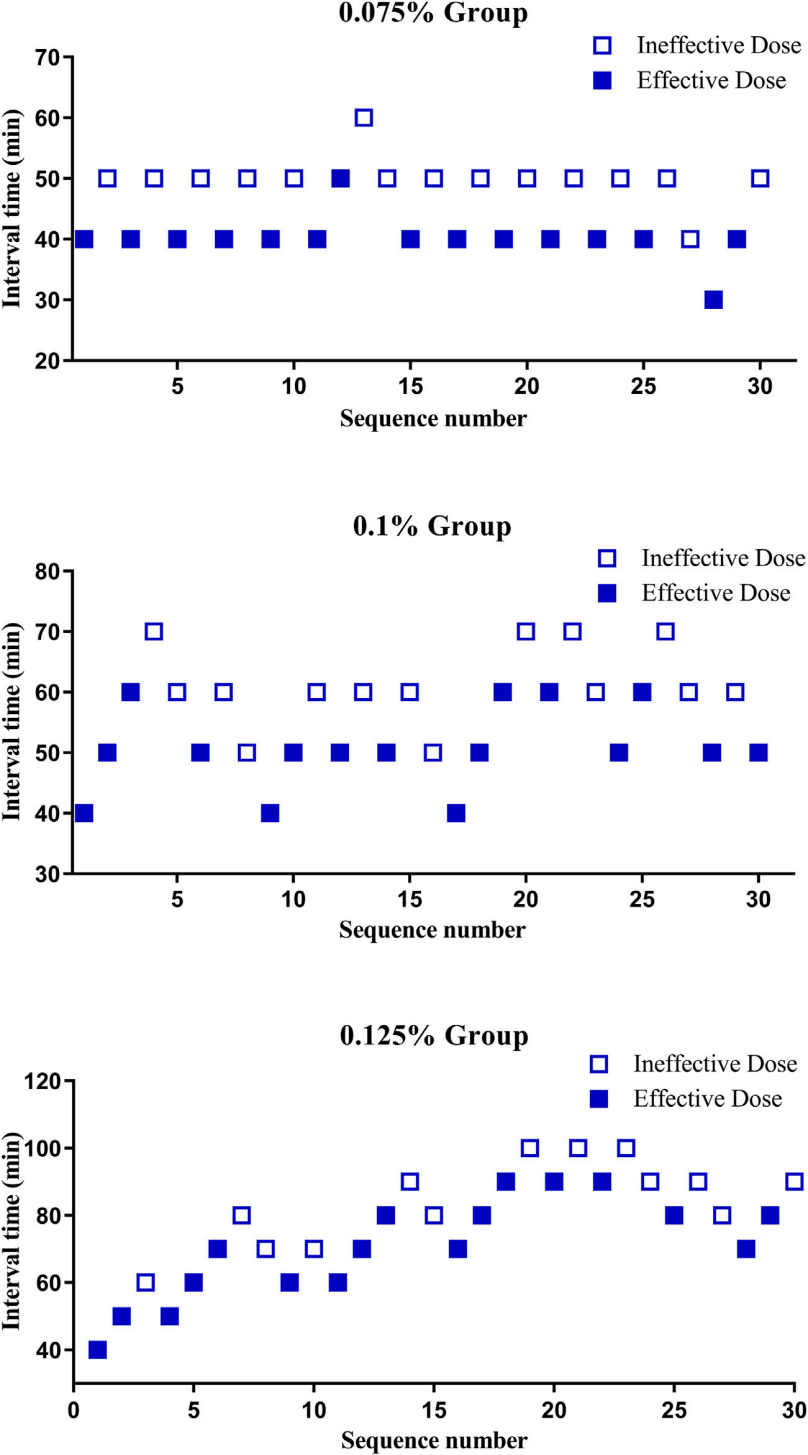
The sequence of programmed intermittent epidural boluses (PIEB) interval times in each group.

The sequence of PIEB interval times is illustrated in Figure 2. The results of isotonic regression analysis at different concentrations indicated that the ED90 values for PIEB interval times were 40.3 (95% CI, 38.3–42.8) minutes at a concentration of 0.075%, 47.3 (95% CI, 44.3–50.5) minutes at a concentration of 0.1%, and 53.9 (95% CI, 49.8–58.3) minutes at a concentration of 0.125%.

The maternal and neonatal outcomes were comparable among the groups, as shows in Table 2.

**TABLE 2:** Maternal and neonatal outcomes.

	0.075% group (n = 30)	0.1% group (n = 30)	0.125% group (n = 30)	*P* value
Analgesic duration, min	239.00 ± 110.27	258.00 ± 151.14	261.73 ± 101.73	0.745
Baseline VAS	8 [7, 9]	8 [7, 9]	8 [6, 9]	0.417
VAS ≤1 within 10 min, n (%)	11 (36.67)	17 (56.67)	16 (53.33)	0.252
Time to VAS ≤1 after the inital analgesia	14.90 ± 8.01	11.76 ± 6.17	11.77 ± 5.64	0.119
Sensory block				
Left	8 [8, 9]	8 [8, 8]	8 [8, 8]	0.238
Right	8 [8, 9]	8 [8, 8]	8 [8, 8]	0.297
Highest	8 [7, 8]	8 [7, 8]	7 [6, 8]	0.651
T6, n (%)	3 (10.00)	7 (23.33)	8 (26.67)	0.233
Asymmetric block, n (%)	0 (0.00)	0 (0.00)	0 (0.00)	>0.999
S1 block, n (%)	30 (100.00)	30 (100.00)	30 (100.00)	>0.999
S2 block, n (%)	30 (100.00)	30 (100.00)	30 (100.00)	>0.999
Rescue bolus	0 (0.00)	1 (3.33)	0 (0.00)	>0.999
Rescue PCEA	0 (0.00)	0 (0.00)	0 (0.00)	>0.999
Bromage score				0.051
0	29 (96.67)	27 (90.00)	23 (76.67)	
1	1 (3.33)	3 (10.00)	7 (23.33)	
*Postartum* headache, n (%)	0 (0.00)	0 (0.00)	0 (0.00)	>0.999
Cesarean delivery, n (%)	3 (10.00)	3 (10.00)	2 (6.67)	0.652
Apgar score (1 min)	10 [9, 10]	10 [9, 10]	10 [9, 10]	0.903
Apgar score (5 min)	10 [10, 10]	10 [10, 10]	10 [10, 10]	>0.999
Patient Satisfaction				0.227
5	25 (83.33)	29 (96.67)	27 (90.00)	
4	5 (16.67)	1 (3.33)	3 (10.00)	

Values are n (%), mean ± SD, or median (IQR).

## 4 Discussion

The findings of this randomized sequence allocation study suggest that the utilization of 0.075%, 0.1%, and 0.125% ropivacaine in combination with PIEB and DPE techniques for labor analgesia results in optimal PIEB interval times of 40.9 (95% CI, 35.3–45.8), 45.3 (95% CI, 39.3–51.5), and 52.9 (95% CI, 46.8–59.3) minutes, respectively.

Zhou et al. (2020) utilized 10 mL of 0.08% bupivacaine combined with 0.3 μg/mL sufentanil to perform PIEB using the sequence allocation method, with a gradient duration of 10 min and a range between 30–60 min. The isotonic regression analysis determined the ED90 of PIEB interval time to be 39.5 (95% CI, 32.5–50.0) minutes. Mei et al. (2024) employed a lower concentration of ropivacaine at 0.0625%, combined with 0.4 μg/mL dexmedetomidine, totaling to an administered volume of 10 mL for PIEB administration in patients who received random interval times ranging from 40, 50, 60 and up to 70 min. The ED90 of PIEB interval time was found to be 45.4 (95% CI, 35.5–50.5) minutes through probit regression analysis. The results obtained from both studies demonstrated a similar ED90 required for maintaining the desired PIEB interval times compared to our study, which utilized a concentration of bupivacaine at 0.075% and an approximate total volume close to 10 mL (40.9 [95% CI, 35.3–45.8] minutes).

Song et al. (2021) used 8 mL of 0.1% ropivacaine combined with 0.3 μg/mL sufentanil PIEB for sequence allocation method, and found that the ED90 of PIEB interval time was 40.5 (95% CI, 33.7–47.5) minutes by isotonic regression analysis. Yao et al. (2023) conducted a randomized trial with 5-min interval times ranging from 35 to 55 min, administering 10 mL of 0.1% ropivacaine combined with 0.3 μg/mL sufentanil and determined the ED90 to be 37.0 (95% CI, 28.4–40.9) minutes. Our study found an ED90 of 45.3 (95% CI, 39.3–61.5) minutes using a higher dose of PIEB (8 mL vs. Song et al. (2021) 10 mL), which may account for the longer duration compared to the aforementioned studies. Xiao et al. (2023) investigated the optimal volume of PIEB by combining PIEB with DPE technology, revealing that the ED90 for ropivacaine at a concentration of 0.1% was 11.3 mL, achieving success rates of 64% and 76% at volumes of 8 mL and 10 mL respectively. Ran et al. (2022) determined that the optimal volumes for PIEB with ropivacaine concentrations of 0.075% and 0.1% were found to be 10 mL and 9 mL, respectively. This corresponds to hourly doses of ropivacaine administered at rates of either 11.25 mg/h or 13.5 mg/h accordingly, resulting in lower incidence rates for motor blockade and adverse events. The utilization of a 10 mL PIEB volume and an additional top-up volume of 30 min after the initial loading may potentially account for our prolonged PIEB interval time. Furthermore, in their study, Yao et al. (2023) employed a randomized trial and isotonic regression analysis to determine the optimal PIEB interval time. It should be noted that the up-and-down sequence allocation method is more appropriate for isotonic regression analysis compared to probit regression analysis as it yields a more accurate ED90 value and narrower 95% CI.

Shi et al. (2024) conducted a study comparing the analgesic effects of different concentrations (0.075%, 0.1%, and 0.125%) of ropivacaine combined with 0.5 μg/mL sufentanil for labor analgesia, which revealed that increasing the concentration resulted in better overall analgesic effect, longer duration of analgesia, and a longer time to first PCEA; however, it also led to higher Bromage scores. The study concluded that maintenance interval time increases as the concentration of ropivacaine used increases, with ED90 extended by 4.4 and 12 min for concentrations of 0.1% and 0.125%, respectively, compared to 0.075%, representing an extension ratio of approximately 9.7% and 29%. The combination of a concentration of 0.1% with 0.5 μg/mL sufentanil in Boselli et al. (2003) demonstrated similar efficacy to a ropivacaine concentration of 0.15% in controlling maternal pain and improving maternal satisfaction during labor analgesia. However, despite the smaller overall dose of ropivacaine at low concentrations, there was no reduction in the incidence of motor blockade and adverse events observed. A meta-analysis comparing high concentration (>0.1%), low concentration (0.08%–0.1%), and ultra-low concentration (≤0.08%) for labor analgesia revealed comparable rates for conversion to cesarean section, VAS pain scores at 30 or 60 min, and maternal satisfaction across different concentrations (Halliday et al., 2022b).

In this study, we administered the PIEB combined with DPE technique for lobar analgesia. Maeda et al. (2024) discovered that utilizing the DPE technique resulted in a 35% reduction in the initial bupivacaine dosage compared to traditional epidural analgesia (29.30 vs. 45.25 mg) through a sequence allocation method. This has significant advantages for both the patients and newborns, including improved patient comfort and satisfaction due to rapid onset, as well as reduced exposure to local anesthetics by subsequently decreasing the cumulative amount of epidural local anesthetic used, thereby lowering the potential risk of adverse events such as motor blockade and maternal hypotension. Similar benefits were also demonstrated in studies involving PIEB combined with DPE technique (Song et al., 2021). The meta-analysis conducted by Wydall et al. (2023) demonstrated that compared to continuous epidural lobar analgesia, PIEB effectively reduced pain scores at 2 and 4 h post-analgesia, decreased the overall dosage of local anesthetics and incidence of motor blockade, and enhanced maternal satisfaction. Furthermore, Howle et al. (2024) meta-analysis revealed that PIEB administered at intervals of 10 mL every 60 min or 5 mL every 30 min was the optimal protocols for reducing the total dose of local anesthetics and minimizing motor blockade occurrence. These findings provide additional support for the results obtained in this study.

The results of the meta-analysis indicated that patients in the low-concentration group had approximately twice the likelihood of obtaining a 1-min Apgar score <7 compared to those in the high-concentration and ultra-low-concentration groups, which was attributed to increased usage of epidural opioids (Halliday et al., 2022a). However, our study did not yield similar findings as all newborns exhibited higher Apgar scores. Due to the limited sample size, it was insufficient to observe further changes in Apgar scores. On the contrary, due to the sequential allocation method, the timing of receiving PIEB in different groups was variable, and consequently, the corresponding dosage of local anesthetics and opioid drugs was not standardized. Therefore, it became challenging to further assess the impact of opioids on Apgar score. Moreover, a randomized controlled trial demonstrated that administering low-concentration local anesthetics did not have any adverse effects on neonatal outcomes such as fetal bradycardia, amniotic fluid contamination, incidence of oxygen requirement, and Apgar scores at 1 and 5 min (Baliuliene et al., 2018).

There are several limitations in this study. We observed both primiparous and multiparous women; however, it should be noted that multiparous women exhibited faster delivery times and lower pain scores compared to primiparous women. This discrepancy could potentially influence our assessment of the optimal PIEB interval time and its impact on analgesic scores. Additionally, we solely focused on including women with cervical dilation <5 cm; thus lacking guidance for those with greater cervical dilation. Lastly, our study only examined three different concentrations of local anesthetics without considering higher concentrations; consequently affecting the future clinical applicability of these findings.

In conclusion, when PIEB is combined with DPE for labor analgesia, the optimal PIEB interval times for 0.075%, 0.1%, and 0.125% concentrations of ropivacaine were determined to be 41, 45, and 53 min respectively. The interval times for 0.1% and 0.125% ropivacaine concentrations were found to be extended by approximately 9.7% and 29% respectively compared to that of the concentration of 0.075%.

## Data Availability

The original contributions presented in the study are included in the article/supplementary material, further inquiries can be directed to the corresponding authors.
